# Does Protein Ingestion Timing Affect Exercise-Induced Adaptations? A Systematic Review with Meta-Analysis

**DOI:** 10.3390/nu17132070

**Published:** 2025-06-21

**Authors:** Rafael A. Casuso, Lennert Goossens

**Affiliations:** 1 Department of Health and Biomedical Sciences, Universidad Loyola Andalucía, Calle Escritor Castilla Aguayo, 4, Poniente Sur, 14004 Córdoba, Spain; 2 Department of Health and Biomedical Sciences, Universidad Loyola Andalucía, Avenida de las Universidades, 2, 41704 Sevilla, Spain; lgoossens@uloyola.es

**Keywords:** resistance training, supplementation timing, chronobiology, strength, body composition

## Abstract

Background/Objectives: Muscle strength and mass are key determinants of exercise performance and a hallmark of health span. Recently, several meta-analyses have concluded that protein supplementation timing does not alter muscle strength and mass gains. However, these meta-analyses did not directly compare several supplementation timings within the same study, thus limiting their conclusions. The objective of this study was to conduct a meta-analysis including only studies directly comparing protein intake before and after exercise. Methods: Three databases (PubMed (*n* = 748), Web of Science (*n* = 1458), and Scopus (*n* = 1105)) and reference lists were searched from inception to January 15, 2024 to identify studies where subjects were randomized to consume protein before or after each training session for at least 4 weeks. Risk of bias was evaluated using the critical appraisal checklist for RCT. A meta-analysis was performed using random-effect models. The outcomes were strength and lean body mass. Results: Of 3311 records identified, 6 reports (5 studies) were eligible and all were considered of sufficient quality to be included in the meta-analysis. For the chest press exercise, there was no effect of protein timing on repeated maximum (RM) (SMD: 0.07; 95% CI: −0.248 to 0.395; *I*^2^ = 0%, *p* = 0.653). For the leg press exercise, consuming protein before training increased the RM more than after training (SMD: 0.70; 95% CI: 0.005 to 1.388; *I*^2^ = 31%, *p* = 0.048). However, subgroup analysis did not reveal a significant effect difference (*p* = 0.07) for leg press and chest press. Lean body mass was not differently modulated by protein supplementation timing (SMD: −0.08; 95% CI: −0.398 to 0.244; *I*^2^ = 0%, *p* = 0.641). Conclusions: Protein timing does not importantly modify exercise-induced changes in lean body mass. While upper and lower limbs strength may respond differently, more investigation is needed to reach a more robust conclusion. The present review was registered in PROSPERO (CRD42023464503).

## 1. Introduction

Skeletal muscle contains the largest portion of the body’s protein content. While muscle proteins change much more slowly than those in other tissues [1,2], skeletal muscle contributes the most to overall protein turnover in the body due to its large protein mass [1]. Current protein intake recommendations suggest 0.8 g/kg of body weight for young healthy subjects, which can be increased to approximately 1.2 g/kg of body weight in older subjects and to approximately 1.6–1.8 g/kg in athletes [3,4,5]. Supplementation is an effective method to ensure the intake of sufficient protein for enhancing skeletal muscle strength and mass in healthy adults and older people [6,7]. These increases in skeletal muscle strength and mass can help to improve athletic performance and health span. It has been described that whey protein supplementation is more effective than casein or soy protein in stimulating muscle protein synthesis after exercise [8], likely due to its higher leucine content, which is known to stimulate the mechanistic target of rapamycin complex−1 (mTORC1), a key protein that triggers muscle protein synthesis.

During the last few years, a growing number of studies have shown that adaptations to exercise can be time-of-the-day dependent [9]. Moreover, it has been recently demonstrated in rodents that the molecular regulators of muscle protein synthesis show a circadian rhythmicity [10]. In humans, ingesting branched-chain amino acids (BCAA) immediately before intense leg exercise increases the anabolic response compared to the consumption of these amino acids immediately after [11]. So far, there is no conclusive evidence regarding protein supplementation timing around exercise to maximize skeletal muscle strength and mass.

It has been suggested that the ingestion of protein either before and/or after exercise can be an effective strategy to support increases in strength and improvements in body composition [12], but this effect likely diminishes as more time passes after the exercise session [13]. Schoenfeld et al. [14] concluded from their meta-analysis that the timing of protein intake in and around a training session does not influence muscular adaptations. However, as Beale [15] rightfully commented, the studies chosen for analysis were not suitable to draw this conclusion, because most of them compared protein supplement to placebo and did not match total daily protein intake between groups.

Various systematic reviews and meta-analysis have been conducted to address this protein timing question. Wirth et al. [16] found no significant differences in muscle mass and strength among groups receiving protein supplementation after exercise, both before and after exercise, or not around exercise. However, a direct comparison between protein supplementation before and after exercise was not included. Moreover, results by Zhou et al. [17] indicated a more beneficial effect of protein intake after compared to before exercise for improving muscle mass and strength, but only one study directly comparing protein intake before and after exercise was included. Consequently, the limitations as commented by Beale [15] were not resolved.

To optimize muscle benefits in individuals following a resistance training (RT) program, protein supplementation timing should be thoroughly investigated. To date, evidence on the effects of protein supplementation before and after exercise is inconclusive. Therefore, the purpose of this paper is to conduct a meta-analysis including only studies directly comparing protein intake before and after exercise.

## 2. Materials and Methods

The protocol was registered in the International Prospective Register of Systematic Reviews (PROSPERO) database (CRD42023464503). The present review followed the guidelines from the Preferred Reporting Items for Systematic Reviews and Meta-Analyses (PRISMA) 2020 statement [18]. The PRISMA 2020 checklist (Supplementary Table S1) indicates where the information can be found.

### 2.1. Literature Search Strategy

We scrutinized three databases (PubMed, Web of Science (WOS), and Scopus) to search for eligible studies from inception to 15 January 2024. In addition, reference lists were examined (references cited in study reports included in the systematic review as well as references cited in systematic review reports on the same or a similar topic) until 26 January 2024. The following search strategy was used in all three databases: (“protein supplementation” OR “protein intake” OR “nutritional supplementation” OR “protein supplement” OR “nutritional supplement” OR “food intake” OR “protein” OR “nutrient”) AND (“exercise” OR “training” OR “sport”) AND (“timing” OR “anabolic window” OR “time of day” OR “time-of-day” OR “time of the day” OR “time-of-the-day”). In Web of Science, the search was limited to the Core collection. In Scopus, the search was limited to article title, abstract, and keywords. The ‘Systematic Review Accelerator Deduplicator’ (https://sr-accelerator.com/#/deduplicator, accessed on 10 February 2024) was used to remove duplicates.

### 2.2. Selection Criteria and Data Extraction

We aimed to identify randomized controlled trials where a training protocol was applied at least twice a week for at least 4 consecutive weeks and where one group was supplemented with protein before and another group after each training. The main outcomes that must be included in each study are those related to strength and body composition.

The selection of the studies was independently performed by the two researchers. In the case that the study abstract suggested that the study could meet the inclusion criteria, the full text was retrieved and carefully assessed. Discrepancies during the study selection were resolved by consensus. The inclusion criteria were as follows: (1) randomized controlled trials or crossover studies with at least 4 weeks of washout; (2) adult subjects (>18 years); (3) supplementation window (from 4 h pre-exercise to 4 h post exercise); and (4) consumption of protein beverages (e.g., whey) or branched chain amino acids (BCAA). Exclusion criteria were: (1) animal studies; (2) acute studies; (3) timing outside the proposed supplementation window; and (4) protein co-ingested with other potentially hypertrophic agents.

Relevant predetermined variables were extracted independently by the two authors of the study. Discrepancies during the data extraction process were resolved by consensus. These variables included study population size; study participants’ health status, age, weight, height, and BMI; study design, details of the training, details of the supplementation, subjects’ characteristics, any strength outcomes, and any body composition outcomes, as well as instruments used for strength and body composition assessment. For the lean mass meta-analysis, we also included the data of fat-free mass provided by Wycherley et al. [19]. In the context of our meta-analysis, this allows us to capture the broader impact on body composition. If necessary, the study investigators were contacted by e-mail to obtain missing variables.

### 2.3. Quality Assessment

The two researchers independently evaluated risk of bias using the critical appraisal checklist for RCT of the Faculty of Health and Medical Sciences at the University of Adelaide, South Australia [20]. The checklist consists of 13 items related to randomization, blinding, group similarity, follow-up, outcome measurement, statistical analysis, and trial design. Each item was scored with ‘yes’, ‘no’, ‘unclear’, or ‘not applicable’, and for each article an overall appraisal (include or exclude) was made. Any disagreements were resolved through discussion.

### 2.4. Meta-Analysis Procedures

All the analyses were performed using the metafor package of R software13. Meta-analyses were performed using random-effect models with DerSimonian–Laird methods to evaluate the effect of supplementation timing on strength and body composition. The change in mean and change in standard deviation (ΔSD) were recorded for each timing (i.e., before and after exercise) and outcome. When ΔSD was not reported, it was calculated assuming a correlation coefficient of 0.7 as previously suggested [21,22]:

$$\Delta SD=\sqrt{\left\{SD_{\left\{pre\right\}}^{2}+SD_{\left\{post\right\}}^{2}-2\times corr\times SD_{\left\{pre\right\}}\times SD_{\left\{post\right\}}\right\}}$$

As the results of the studies were not reported in comparable scales, effect sizes are presented as standardized mean difference (SMD) and 95% confidence intervals (CI_s_).

Heterogeneity was reported as the *I*^2^ value and the prediction interval derived from Tau and Cochran Q. Following the Cochrane Handbook recommendations, heterogeneity (*I*^2^) was classified as low (0–40%), moderate (40–75%), or high (75–100%). In addition, to assess whether any single study disproportionately influenced the overall effect size, we performed a leave-one-out sensitivity analysis, sequentially removing one study at a time and recalculating the pooled effect estimate. Risk of bias was assessed using visual inspection of Funnel plots and accompanying Egger’s Tests using the metafor package. The certainty of evidence was graded following the GRADE assessment [23]. The body of evidence began with a high-certainty rating and was lowered by one level for each GRADE domain that presented severe problems.

## 3. Results

**Figure 1:**
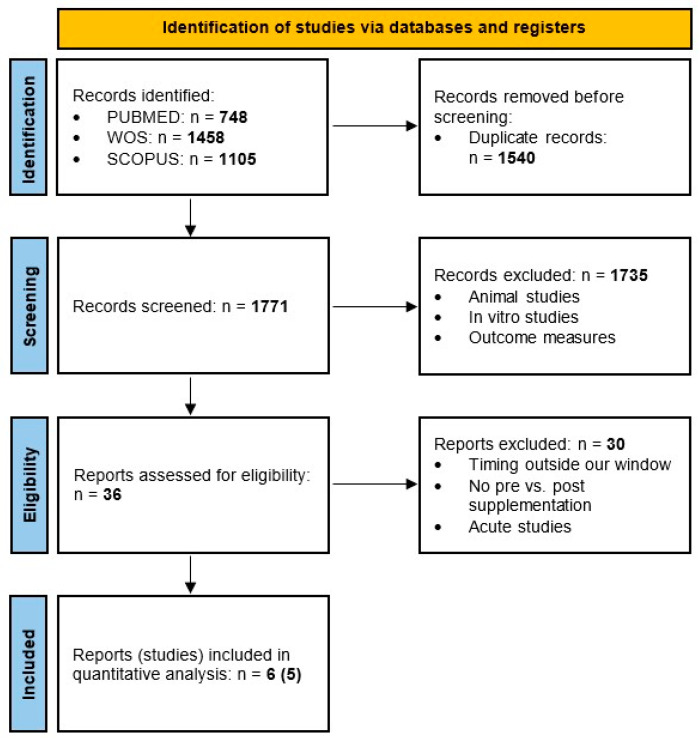
PRISMA flowchart.

The original search yielded 3311 studies (WOS = 1458; SCOPUS = 1105; PUBMED = 748) and a total of 1771 were screened after duplicates were removed. After selection of the studies according to the eligibility criteria, six reports (five different studies) were included in the meta-analysis. No extra articles that fulfilled inclusion criteria were found from reference list examination. A PRISMA flowchart can be found in Figure 1.

All studies were randomized controlled trials. One study [24] involved young, trained men and one study [25] young, trained women. One study [26] involved older, untrained men and two others [27,28] older, untrained women. One study [27] involved older, overweight or obese men and women with type 2 diabetes. One study [27] scores 100% on the critical appraisal checklist, one study [26] scores 92%, two studies [19,25] score 62%, and one study [24] scores 46%. All studies were considered of sufficient quality to be included in the meta-analysis. The risk of bias assessment can be found in Table 1.

A summary of the study characteristics can be found in Table 2. As the protein supplementation source, whey protein was used in three studies [24,27,28] and milk protein in one study [19]. Another study [26] used a combination of whey, milk, and egg protein, whereas the last study [25] did not report the protein source. One study [26] individualized the amount of protein (0.3 g/kg), and in the other studies [19,24,25,26,27,28], the amount of protein varied from 25 to 27.1 g. Protein supplementation timing was immediately before versus immediately after resistance training in all studies except one [19]. In the latter study, supplementation immediately before was compared to supplementation 2 h after resistance training. To control for differences in total protein and caloric intake, all studies applied dietary intake reporting. Additionally, two studies prescribed a diet concomitant with the supplementation intervention—one moderately energy-restricted diet [19] and one diet to create an energy surplus of 500 kcal/day [24]. Four studies [25,26,27,28] reported no differences in caloric or protein intake between groups. One study [26] reported more protein consumption before training in the ‘before’ group compared to the ‘after’ group.

All studies used progressive whole-body resistance training on non-consecutive days. Four studies [19,24,26,27,28] applied three training sessions per week and one study [25] applied two. Duration of the training period was six [25], ten [24], twelve [26,27,28], or sixteen [19] weeks.

All studies evaluated outcome measures related to maximum strength and body composition. Regarding strength, five studies measured chest press 1RM. In addition, two studies [25,26] measured leg press 1RM, one study [19] lat pull-down 1RM, one study [24] back squat 1RM, and one study [19] knee extension 1RM and preacher curl 1RM. Regarding body composition, four studies [24,26,28] compared lean body mass, one study [19] compared fat-free mass, and four studies [19,24,25,28] compared fat mass. Two studies [24,26] compared biceps, triceps, and knee extensor muscle thickness.

**Figure 2:**
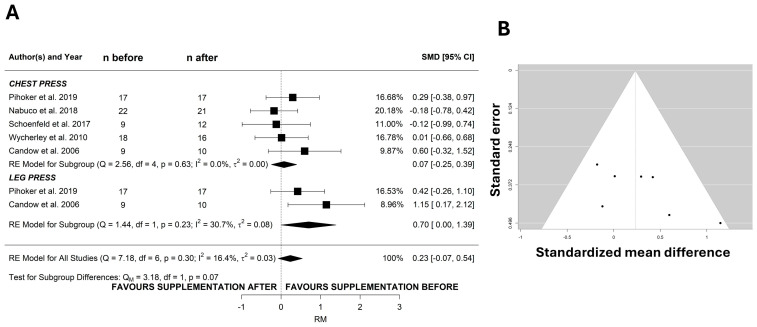
Protein ingestion before resistance training increases leg strength but not upper body strength. Forest plot of the effects of protein timing on RM (**A**). Panel (**B**) shows the funnel plots for RM. RE—random effect; RM—one repetition maximum; SMD—standardized mean difference [19,24,25,26,27].

The change in chest press RM was assessed in five studies, of which two also reported the changes in leg press RM (Figure 2A). The overall effect for chest press showed no effect of protein timing (SMD: 0.07; 95% CI: −0.248 to 0.395; *I*^2^ = 0%, *p* = 0.653, *n* = 5 studies). On the other hand, consuming protein before training induced a higher leg press RM gain than consuming protein after training (SMD: 0.70; 95% CI: 0.005 to 1.388; *I*^2^ = 31%, *p* = 0.048, *n* = 2 studies). However, subgroup analysis did not reveal a significant effect difference (*p* = 0.07) for leg press and chest press. The funnel plot did not show evident asymmetries (Figure 2B) and the Egger test was not significant (*p* = 0.104). The sensitivity analysis reported that none of the included studies (*n* = 7) influenced the overall effect shown in Figure 2A.

**Figure 3:**
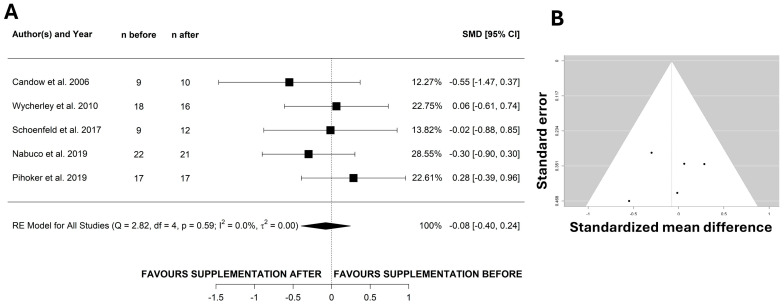
Protein ingestion timing does not affect lean mass. Forest plot of the effects of protein timing on lean mass (**A**). Panel (**B**) shows the funnel plots for lean mass. RE—random effect; SMD—standardized mean difference [19,24,25,26,28].

Total body lean mass was assessed in five studies (Figure 3A). The meta-analysis revealed that the timing of protein consumption did not affect total body lean mass (SMD: −0.08; 95% CI: −0.398 to 0.244; *I*^2^ = 0%, *p* = 0.641, *n* = 5 studies). The funnel plot did not show asymmetries (Figure 3B) and the Egger Test was not significant (*p* = 0.684). The sensitivity analysis reported that none of the included studies significantly influenced the overall effect. A similar lack of effect for protein timing was observed in studies analyzing fat mass changes (Supplementary Figure S1). Finally, bicep (SMD: −0.197; 95% CI: −0.826 to 0.432; *I*^2^ = 0%, *p* = 0.540, *n* = 2 studies), vastus lateralis (SMD: 0.128; 95% CI: −0.658 to 0.914; *I*^2^ = 36%, *p* = 0.749, *n* = 2 studies), and triceps (SMD: 0.314; 95% CI: −0.371 to 0.999; *I*^2^ = 15%, *p* = 0.369, *n* = 2 studies) thickness were not influenced by protein timing (Supplementary Figure S2).

The evidence was downgraded two steps because of risk of bias and imprecision for the outcomes for chest press, lean mass, and fat mass. The certainty of evidence for these outcomes was valued as ‘low’. The evidence was downgraded three steps because of risk of bias, imprecision, and because several studies included in the review did not contribute to the outcomes for leg press, bicep thickness, triceps thickness, and vastus lateralis thickness. The certainty of evidence for these outcomes was valued as ‘very low’ (Supplementary Table S2). A summary of the findings can be found in Supplementary Table S3.

## 4. Discussion

In the present study, we performed a meta-analysis of randomized controlled trials to quantitatively assess the effects of consuming protein before and after training on strength gains and body composition. To the best of our knowledge, this is the first study synthesizing this data from RCTs, as previous meta-analysis pooled data from studies that were not designed to compare protein supplementation before and after training [14,15]. It should, however, be noted that the findings from the present study are in accordance with those from the previously mentioned meta-analyses, as we found that strength gains and lean body mass changes are not influenced by protein timing. Notably, subgroup analysis reveals that supplementation before training can increase leg press RM to a higher extent than supplementation after training.

The observation that pre-exercise protein supplementation may enhance leg strength more than post exercise should be carefully interpreted. Leg press assessment was only performed in two studies [24,25] involving 53 subjects. In addition, the subgroup comparison for leg press and chest press showed only a statistical trend. Nevertheless, the possibility that different anatomical regions can respond in a different manner to protein timing needs to be addressed in future studies.

It has been previously suggested that pre-exercise protein supplementation can prevent protein breakdown and acts as exogenous fuel [29]. Protein breakdown is evident during prolonged endurance exercise [30], while evidence during resistance exercise is lacking. In contrast, it is well known that skeletal muscle mitochondria are the main site for BCAA catabolism [31], and legs have higher mitochondrial content than arms [32]. Therefore, enhancing fuel availability by pre-exercise supplementation may have a greater impact on leg performance. In this regard, it should be noted that the two studies assessing leg press used training protocols with fixed repetitions. In the described scenario with fixed repetitions and enhanced fuel source for the legs, the legs might be more separated from failure than the arms. One inevitable consequence of approaching failure is a progressive decrease in velocity. Notably, a period of resistance training with low velocity loss (25%) increases maximal strength to a higher extent than training with high velocity loss (50%) [33]. Therefore, one hypothesis that would need to be tested is whether pre-exercise protein supplementation alters the force–velocity profile compared to post exercise supplementation. Additionally, it would be interesting to test protein oxidation capacity in different anatomical regions (i.e., arms vs. legs).

It should be noted that our pooled analysis included data from RCT studies where differences in energy intake between experimental groups (i.e., before and after) was controlled for, solving one of the issues highlighted by Beale [15]. Moreover, previous meta-analyses have compared the effect size of studies differing in supplementation timing [14,16,17]. This kind of analysis limits conclusions, as no direct comparison of the timing effect–size was computed within each study. To solve this issue, we searched for RCT studies where one group was supplemented before, and another group was supplemented after each training session. Our conclusions from this robust analysis are in accordance with the previously mentioned meta-analyses. However, this approach resulted in a limited number of studies included in the present analysis. Because of this limitation, we have been unable to perform meta-regressions of key variables such as age or training status of the participants, training length, or supplementation dose. To reach stronger conclusions, we, therefore, recommend researchers to perform an RCT comparing protein supplementation before and after training and to assess different populations.

The results of this study should be interpreted with caution due to several limitations. First, only two of the included studies received a “good” rating in the risk of bias assessment, indicating that the methodological quality of most of the studies analyzed is limited. Second, some of the meta-analyses conducted in the present study were based on only two observations, which, combined with the aforementioned issue, further restricts the strength of the conclusions. To enhance the scientific rigor of our findings, we also performed a GRADE assessment of the certainty of the evidence. This evaluation rated the certainty for each outcome as low to very low. Nevertheless, the in-depth assessment of these limitations represents a strength of our work, as it underscores the need for methodologically rigorous research to determine the effects of protein intake timing—before or after exercise—on strength and body composition outcomes.

Taking these limitations into account, we conclude that protein supplementation within the time window of 15 min pre-exercise to approximately 2 h post exercise does not significantly affect muscle strength and body composition. However, consuming protein within 15 min before exercise might help improve leg strength. Furthermore, it remains unclear whether exercise adaptations can be modulated by protein timing in different populations.

## Figures and Tables

**Table 1:** Critical appraisal checklist evaluation of studies analyzing protein timing on exercise-induced muscular adaptations.

	True Randomization	Allocation Concealed	Group Similarity at Baseline	Blinded Participants	Blinded Treatment Deliverers	Blinded Outcomes Assessors	Identically Treated Groups	Follow up Complete/Adequately Described and Analyzed	Participants Analyzed in Randomized Groups	Same Way of Outcome Measurement for Treatment Groups	Reliable Outcome Measurement	Appropriate Statistical Analysis	Appropriate Trial Design/Deviations Considered in the Conduct and Analysis	Overall Appraisal
Candow et al., 2006 [26]	Unclear	Yes	Yes	Yes	Yes	Yes	Yes	Yes	Yes	Yes	Yes	Yes	Yes	Include
Wycherley et al., 2010 [19]	Unclear	Unclear	Yes	No	Unclear	Unclear	Yes	Yes	Yes	Yes	Yes	Yes	Yes	Include
Schoenfeld et al., 2017 [24]	Unclear	Unclear	Unclear	No	Unclear	Unclear	Yes	No	Yes	Yes	Yes	Yes	Yes	Include
Nabuco et al., 2018 [27] Nabuco et al., 2019 [28]	Yes	Yes	Yes	Yes	Yes	Yes	Yes	Yes	Yes	Yes	Yes	Yes	Yes	Include
Pihoker et al., 2019 [25]	Unclear	Unclear	Yes	No	Unclear	Unclear	Yes	Yes	Yes	Yes	Yes	Yes	Yes	Include

**Table 2:** Summary of studies analyzing protein timing on exercise-induced muscular adaptations meeting inclusion criteria.

Study	Subjects	Supplement	Supplementation Timing	Protein Matched with Control?	Assessment Methods	Training Protocol	Significant Results on Strength?	Significant Results on Body Composition?
Candow et al., 2006 [26]	29 untrained, older men	Supplement of 0.3 g protein/kg body mass (from whey, milk, egg) (in 0.54 g/kg of Myoplex^®^), and 0.09 g/kg chocolate cocoa.	(1) Protein immediately before and placebo immediately after; (2) placebo immediately before and protein immediately after; (3) placebo immediately before and placebo immediately after.	No. Dietary records for 3 days during the first and final week indicated that the ‘before’ group consumed significantly more dietary protein before training compared to the ‘after’ group.	Air-displacement plethysmography; B-Mode ultrasound; 1-RM	12 weeks of progressively increased resistance training, 3 non-consecutive days/week, 60 min	Chest press 1-RM/leg press 1-RM	Lean tissue mass/muscle thickness
Wycherley et al., 2010 [19]	34 overweight and obese, sedentary men and women with type 2 diabetes	Supplement of 25 g skim milk powder in 250 mL low fat (<1%) milk (total 860 kJ, 21 g protein, 0.7 g fat, 29.6 g carbohydrate).	(1) Protein immediately before; (2) protein at least 2 h after.	Yes. Total energy intake and macronutrient composition were similar between the treatment groups.	Dual X-ray absorptiometry; 1-RM	16 weeks of progressively increased resistance training, 3 non-consecutive days/week, 45 min	Chest press 1-RM/lat pull-down 1-RM	Fat-free mass/ fat mass
Schoenfeld et al., 2017 [24]	21 young male, experienced lifters	Supplement of 25 g protein and 1 g carbohydrate (Iso100 Hydrolyzed Whey Protein Isolate).	(1) Protein immediately before; (2) protein immediately after.	Yes. There was no significant group effect for self-reported calorie intake.	B-mode ultrasound imaging; dual x-ray absorptiometry; 1-RM	10 weeks of progressively increased resistance training, 3 non-consecutive days/week	Chest press 1-RM/back squat 1-RM	Lean tissue mass/muscle thickness/ fat mass
Nabuco et al., 2018 [27]; Nabuco et al., 2019 [28]	66 untrained, older women	Supplement of hydrolyzed whey protein (27.1 g of protein, 5.2 g of carbohydrates, 0.2 g of fat).	(1) Protein immediately before and placebo immediately after; (2) placebo immediately before and protein immediately after; (3) placebo immediately before and placebo immediately after.	Yes. The habitual daily energy and macronutrients intake were not different between groups.	Dual X-ray absorptiometry; 1-RM	12 weeks of progressively increased resistance training, 3 non-consecutive days/week	Chest press 1-RM/preacher curl 1-RM/knee extension 1-RM	Lean body mass/ fat mass
Pihoker et al., 2019 [25]	43 trained, young women	Supplement of 200 kcal (3.5 g fat, 16 g carbohydrates, 25 g lean protein).	(1) Protein within fifteen minutes before; (2) protein within 15 min after; (3) no supplementation.	Yes. Dietary logs demonstrated no differences in kilocalories.	Dual X-ray absorptiometry; 1-RM	6 weeks of progressively increased resistance training, 2 non-consecutive days/week	Chest press 1-RM/leg press 1-RM	Lean body mass/ fat mass

1-RM = one repeated maximum.

## Data Availability

Any data or other materials will be made available upon request to the first author. The data are not publicly available due to time limitations.

## Supplementary Materials

The following supporting information can be downloaded at: https://www.mdpi.com/article/10.3390/nu17132070/s1, Table S1: PRISMA 2020 checklist; Table S2: GRADE assessment; Table S3: Summary of Findings; Figure S1: Forest and funnel plots of the effects of protein timing on fat mass. RE—random effect; SMD—standardized mean difference; Figure S2: Forest and funnel plots of the effects of protein timing on biceps thickness (A), vastus lateralis thickness (B), and triceps thickness (C). RE—random effect; SMD—standardized mean difference.

## Abbreviations

The following abbreviations are used in this manuscript:

BCAA	Branched chain amino acids
RCT	Randomized controlled trial
RM	Repetition maximum
SMD	Standardized mean difference

## References

[1] Lewis S.E., Kelly F.J., Goldspink D.F. (1984). Pre- and post-natal growth and protein turnover in smooth muscle, heart and slow- and fast-twitch skeletal muscles of the rat. Biochem. J..

[2] Goldspink D.F., Kelly F.J. (1984). Protein turnover and growth in the whole body, liver and kidney of the rat from the foetus to senility. Biochem. J..

[3] Phillips S.M., Chevalier S., Leidy H.J. (2016). Protein “requirements” beyond the RDA: Implications for optimizing health. Appl. Physiol. Nutr. Metab..

[4] Phillips S.M., Paddon-Jones D., Layman D.K. (2020). Optimizing Adult Protein Intake During Catabolic Health Conditions. Adv. Nutr..

[5] Kato H., Suzuki K., Bannai M., Moore D.R., Fisher G. (2016). Protein Requirements Are Elevated in Endurance Athletes after Exercise as Determined by the Indicator Amino Acid Oxidation Method. PLoS ONE.

[6] Morton R.W., Murphy K.T., McKellar S.R., Schoenfeld B.J., Henselmans M., Helms E., Aragon A.A., Devries M.C., Banfield L., Krieger J.W. (2018). A systematic review, meta-analysis and meta-regression of the effect of protein supplementation on resistance training-induced gains in muscle mass and strength in healthy adults. Br. J. Sports Med..

[7] Kirwan R.P., Mazidi M., García C.R., E Lane K., Jafari A., Butler T., de Heredia F.P., Davies I.G. (2022). Protein interventions augment the effect of resistance exercise on appendicular lean mass and handgrip strength in older adults: A systematic review and meta-analysis of randomized controlled trials. Am. J. Clin. Nutr..

[8] Tang J.E., Moore D.R., Kujbida G.W., Tarnopolsky M.A., Phillips S.M. (2009). Ingestion of whey hydrolysate, casein, or soy protein isolate: Effects on mixed muscle protein synthesis at rest and following resistance exercise in young men. J. Appl. Physiol..

[9] Galan-Lopez P., Casuso R.A. (2023). Metabolic adaptations to morning versus afternoon training: A systematic review and meta-analysis. Sports Med..

[10] Chang S.-W., Yoshihara T., Machida S., Naito H. (2017). Circadian rhythm of intracellular protein synthesis signaling in rat cardiac and skeletal muscles. Biochem. Biophys. Rep..

[11] Tipton K.D., Rasmussen B.B., Miller S.L., Wolf S.E., Owens-Stovall S.K., Petrini B.E., Wolfe R.R. (2001). Timing of amino acid-carbohydrate ingestion alters anabolic response of muscle to resistance exercise. Am. J. Physiol. Endocrinol. Metab..

[12] Kerksick C.M., Arent S., Schoenfeld B.J., Stout J.R., Campbell B., Wilborn C.D., Taylor L., Kalman D., Smith-Ryan A.E., Kreider R.B. (2017). International Society of Sports Nutrition Position Stand: Nutrient timing. J. Int. Soc. Sports Nutr..

[13] Jäger R., Kerksick C.M., Campbell B.I., Cribb P.J., Wells S.D., Skwiat T.M., Purpura M., Ziegenfuss T.N., Ferrando A.A., Arent S.M. (2017). International Society of Sports Nutrition Position Stand: Protein and exercise. J. Int. Soc. Sports Nutr..

[14] Schoenfeld B.J., Aragon A.A., Krieger J.W. (2013). The effect of protein timing on muscle strength and hypertrophy: A meta-analysis. J. Int. Soc. Sports Nutr..

[15] Beale D.J. (2016). Evidence inconclusive-comment on article by Schoenfeld et al. J. Int. Soc. Sports Nutr..

[16] Wirth J., Hillesheim E., Brennan L. (2020). The role of protein intake and its timing on body composition and muscle function in healthy adults: A systematic review and meta-analysis of randomized controlled trials. J. Nutr..

[17] Zhou H.H., Liao Y., Zhou X., Peng Z., Xu S., Shi S., Liu L., Hao L., Yang W. (2024). Effects of timing and types of protein supplementation on improving muscle mass, strength, and physical performance in adults undergoing resistance training: A network meta-analysis. Int. J. Sport Nutr. Exerc. Metab..

[18] Page M.J., McKenzie J.E., Bossuyt P.M., Boutron I., Hoffmann T.C., Mulrow C.D., Shamseer L., Tetzlaff J.M., Akl E.A., Brennan S.E. (2021). The PRISMA 2020 statement: An updated guideline for reporting systematic reviews. BMJ.

[19] Wycherley T.P., Noakes M., Clifton P.M., Cleanthous X., Keogh J.B., Brinkworth G.D. (2010). Timing of protein ingestion relative to resistance exercise training does not influence body composition, energy expenditure, glycemic control or cardiometabolic risk factors in a hypocaloric, high protein diet in patients with type 2 diabetes. Diabetes Obes. Metab..

[20] Barker T.H., Stone J.C., Sears K., Klugar M., Tufanaru C., Leonardi-Bee J., Aromataris E., Munn Z. (2023). The revised JBI critical appraisal tool for the assessment of risk of bias for randomized controlled trials. JBI Evid. Synth..

[21] Viechtbauer W. (2010). Conducting meta-analyses in R with the metafor package. J. Stat. Softw..

[22] Mason S.A., Keske M.A., Wadley G.D. (2021). Effects of vitamin C supplementation on glycemic control and cardiovascular risk factors in people with type 2 diabetes: A GRADE-assessed systematic review and meta-analysis of randomized controlled trials. Diabetes Care.

[23] Schünemann H.J., Higgins J.P.T., Vist G.E., Glasziou P., Akl E.A., Skoetz N., Guyatt G.H., Higgins J.P.T., Thomas J., Chandler J., Cumpston M., Li T., Page M.J., Welch V.A. (2024). Chapter 14: Completing ‘Summary of findings’ tables and grading the certainty of the evidence [last updated May 2025]. Cochrane Handbook for Systematic Reviews of Interventions Version 6.5.1.

[24] Schoenfeld B.J., Aragon A., Wilborn C., Urbina S.L., Hayward S.E., Krieger J. (2017). Pre- versus post-exercise protein intake has similar effects on muscular adaptations. PeerJ.

[25] Pihoker A.A., Peterjohn A.M., Trexler E.T., Hirsch K.R., Blue M.N., Anderson K.C., Ryan E.D., Smith-Ryan A.E. (2019). The effects of nutrient timing on training adaptations in resistance-trained females. J. Sci. Med. Sport.

[26] Candow D.G., Chilibeck P.D., Facci M., Abeysekara S., Zello G.A. (2006). Protein supplementation before and after resistance training in older men. Eur. J. Appl. Physiol..

[27] Nabuco H.C.G., Tomeleri C.M., Sugihara Junior P., Fernandes R.R., Cavalcante E.F., Antunes M., Ribeiro A.S., Teixeira D.C., Silva A.M., Sardinha L.B. (2018). Effects of whey protein supplementation pre- or post-resistance training on muscle mass, muscular strength, and functional capacity in pre-conditioned older women: A randomized clinical trial. Nutrients.

[28] Nabuco H., Tomeleri C., Junior P.S., Fernandes R., Cavalcante E., Venturini D., Barbosa D., Silva A., Sardinha L., Cyrino E. (2019). Effects of pre- or post-exercise whey protein supplementation on body fat and metabolic and inflammatory profile in pre-conditioned older women: A randomized, double-blind, placebo-controlled trial. Nutr. Metab. Cardiovasc. Dis..

[29] Moore D.R., Camera D.M., Areta J.L., Hawley J.A. (2014). Beyond muscle hypertrophy: Why dietary protein is important for endurance athletes. Appl. Physiol. Nutr. Metab..

[30] Dohm G.L., Tapscott E.B., Kasperek G.J. (1987). Protein degradation during endurance exercise and recovery. Med. Sci. Sports Exerc..

[31] Martín J.C.H., Ansa B.S., Álvarez-Rivera G., Domínguez-Zorita S., Rodríguez-Pombo P., Pérez B., Calvo E., Paradela A., Miguez D.G., Cifuentes A. (2024). An ETFDH-driven metabolon supports OXPHOS efficiency in skeletal muscle by regulating coenzyme Q homeostasis. Nat. Metab..

[32] Zinner C., Morales-Alamo D., Ørtenblad N., Larsen F.J., Schiffer T.A., Willis S.J., Gelabert-Rebato M., Perez-Valera M., Boushel R., Calbet J.A. (2016). The physiological mechanisms of performance enhancement with sprint interval training differ between the upper and lower extremities in humans. Front. Physiol..

[33] Rodiles-Guerrero L., Cornejo-Daza P.J., Sánchez-Valdepeñas J., Alcazar J., Rodriguez-López C., Sánchez-Moreno M., Alegre L.M., León-Prados J.A., Pareja-Blanco F. (2022). Specific adaptations to 0%, 15%, 25%, and 50% velocity-loss thresholds during bench press training. Int. J. Sports Physiol. Perform..

